# Longitudinal preclinical evaluation of the novel radioligand [11C]CHDI-626 for PET imaging of mutant huntingtin aggregates in Huntington’s disease

**DOI:** 10.1007/s00259-021-05578-8

**Published:** 2021-10-15

**Authors:** Daniele Bertoglio, Jeroen Verhaeghe, Alan Miranda, Leonie Wyffels, Sigrid Stroobants, Ladislav Mrzljak, Vinod Khetarpal, Mette Skinbjerg, Longbin Liu, Celia Dominguez, Ignacio Munoz-Sanjuan, Jonathan Bard, Steven Staelens

**Affiliations:** 1 Molecular Imaging Center Antwerp (MICA), University of Antwerp, Universiteitsplein 1, Wilrijk, Antwerp, Belgium; 2 μNEURO Research Centre of Excellence, University of Antwerp, Antwerp, Belgium; 3 Department of Nuclear Medicine, Antwerp University Hospital, Edegem, Belgium; 4 CHDI Management/CHDI Foundation, Los Angeles, CA USA

**Keywords:** Neuroimaging, Biomarker, Animal model, mHTT, HD

## Abstract

**Purpose:**

As several therapies aimed at lowering mutant huntingtin (mHTT) brain levels in Huntington’s disease (HD) are currently being investigated, noninvasive positron emission tomography (PET) imaging of mHTT could be utilized to directly evaluate therapeutic efficacy and monitor disease progression. Here we characterized and longitudinally assessed the novel radioligand [^11^C]CHDI-626 for mHTT PET imaging in the zQ175DN mouse model of HD.

**Methods:**

After evaluating radiometabolites and radioligand kinetics, we conducted longitudinal dynamic PET imaging at 3, 6, 9, and 13 months of age (M) in wild-type (WT, *n* = 17) and heterozygous (HET, *n* = 23) zQ175DN mice. Statistical analysis was performed to evaluate temporal and genotypic differences. Cross-sectional cohorts at each longitudinal time point were included for post-mortem [^3^H]CHDI-626 autoradiography.

**Results:**

Despite fast metabolism and kinetics, the radioligand was suitable for PET imaging of mHTT. Longitudinal quantification could discriminate between genotypes already at premanifest stage (3 M), showing an age-associated increase in signal in HET mice in parallel with mHTT aggregate load progression, as supported by the post-mortem [^3^H]CHDI-626 autoradiography.

**Conclusion:**

With clinical evaluation underway, [^11^C]CHDI-626 PET imaging appears to be a suitable preclinical candidate marker to monitor natural HD progression and for the evaluation of mHTT-lowering therapies.

**Supplementary Information:**

The online version contains supplementary material available at 10.1007/s00259-021-05578-8.

## Introduction

Huntington’s disease (HD) is an autosomal dominant neurodegenerative disorder caused by an expanded polyglutamine (CAG) repeat in exon 1 of the huntingtin (*HTT*) gene [1, 2]. This mutated gene encodes mutant huntingtin (mHTT) protein that accumulates and plays a pathophysiological role in neuronal dysfunction, selective neurodegeneration, and forebrain atrophy [3–6] leading to progressive motor, psychiatric, and cognitive impairments in HD patients [7, 8].

Several therapies aimed at lowering mHTT brain levels are currently under clinical evaluation [9, 10], and there is increasing interest in noninvasive objective markers, such as positron emission tomography (PET) imaging of mHTT, that could directly measure mHTT in the living brain. Such tools could directly quantify the therapeutic efficacy of lowering mHTT, overcoming some of the limitations of quantifying mHTT levels in cerebrospinal fluid as a surrogate for cerebral changes that is in current clinical trials [11–14].

A notable property of the mHTT PET radioligands currently under investigation is affinity towards amyloid plaques in post-mortem human tissue that might reduce their specificity [15], and efforts to overcome this issue are currently focused on the development of radioligands with high affinity and selectivity towards mHTT aggregates suitable for clinical applications. We have recently reported the development of a novel high-affinity radioligand, CHDI-626, that targets mHTT and lacks significant affinity towards amyloid plaques, representing a potential candidate for mHTT PET imaging in the clinic [16, 17].

Our objective in this study was to evaluate kinetic modelling properties and to perform a longitudinal quantification of the novel radioligand [^11^C]CHDI-626 for mHTT PET imaging using the zQ175DN knock-in HD mouse model [18, 19]. This model displays hallmarks of mHTT-containing neuropil, soma, and intranuclear inclusions increasing from 3 to 12 months of age (M) [20], thereby allowing assessment of disease progression. We conducted microPET studies in 10 M mice to characterize the in vivo properties of the radioligand. Next, a longitudinal PET imaging of [^11^C]CHDI-626 was performed in mice at 3, 6, 9, and 13 M to monitor the disease course and to assess its ability to discriminate between genotypes and mHTT load for clinical application, including monitoring disease progression and potential application in mHTT-lowering therapeutic interventional trials.

## Material and methods

### Animals

**Fig. 1:**
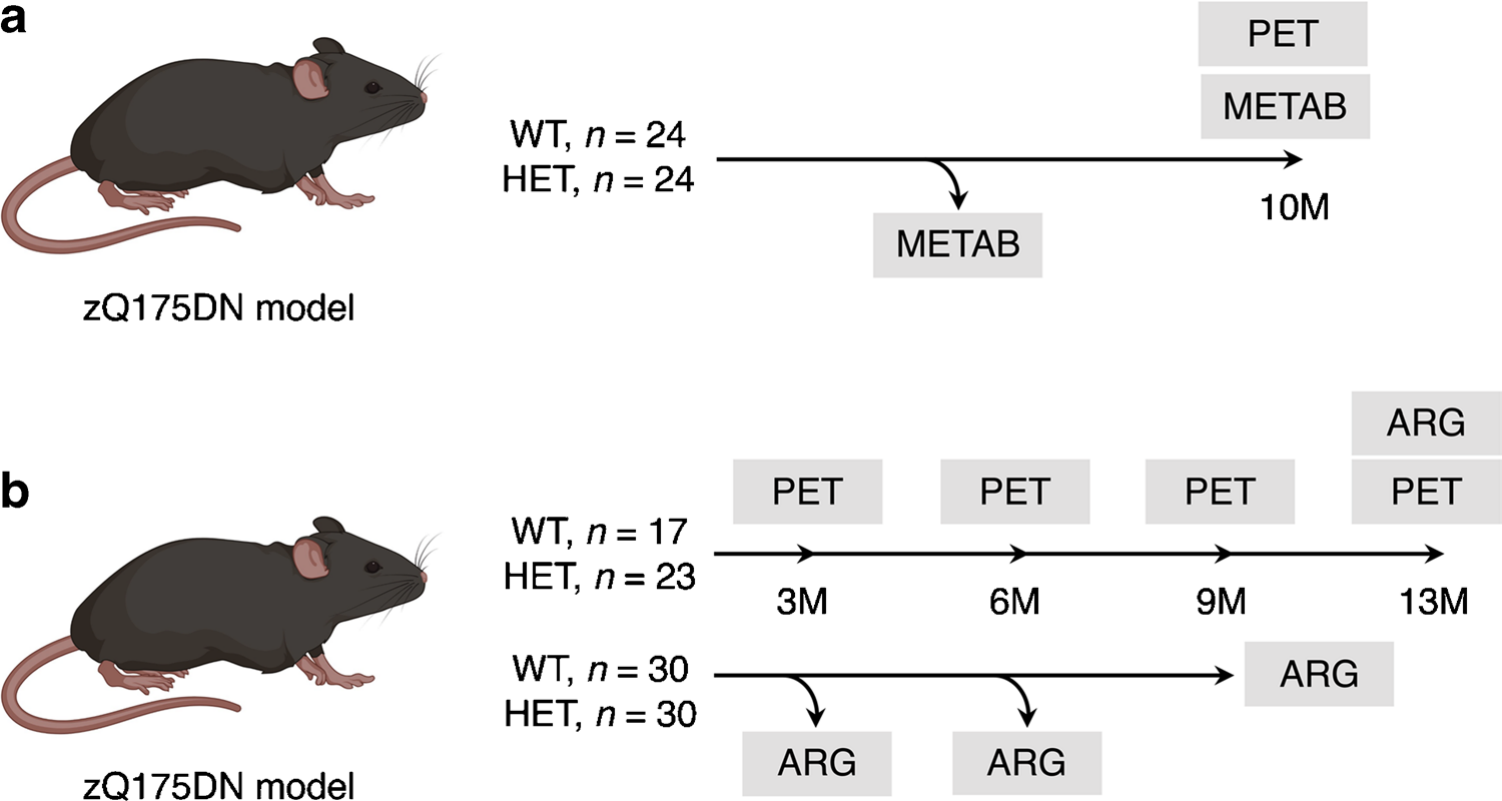
Overview of the experimental design. **a** Number and age of each experimental group included in the kinetic modelling study. **b** Number and age of each experimental group included in the longitudinal and cross-sectional studies. METAB, radiometabolite analysis; ARG, autoradiography

A total of 79 adult male heterozygous (HET, CAG repeats = 193 ± 3.6) and 71 wild-type (WT) littermates zQ175DN knock-in mice (B6J.129S1-Htttm1.1Mfc/190ChdiJ, C57BL/6 J background) were obtained from Jackson Laboratories (Bar Harbour, Maine, USA). A detailed overview of the number and age of the animals allocated to each study is reported in Fig. 1. Animals were single-housed for the longitudinal study and group-housed for cross-sectional studies in individually ventilated cages under a 12-h light/dark cycle in a temperature- and humidity-controlled environment with food and water ad libitum. Single-housing was required to avoid the development of dominant-subdominant and aggressive behaviour. At least 1 recovery week after arrival was allowed before the start of any procedure. Experiments followed the European Committee (decree 2010/63/CEE) and were approved by the Ethical Committee for Animal Testing (ECD 2016–76) at the University of Antwerp (Belgium).

### Radioligand synthesis

[^11^C]CHDI-626 was prepared with an automated synthesis module (CarbonSynthon I, Comecer, Netherlands) via single-step carbon-11 labelling. Thus, a mixture of the pyridone precursor (0.82 mg) in DMSO (0.55 mL) was allowed to react with [^11^C]CH_3_OTf in the presence of Cs_2_CO_3_ (5–6 mg) for 2.5 min at room temperature. Water (0.7 mL) was added to terminate the reaction and to ensure proper retention of the compound of interest on the high-performance liquid chromatography (HPLC) column. The crude product was purified on HPLC using a Kinetex EVO C18, 5 µm, 150 × 10 mm column (Phenomenex, USA), eluted with ethanol (EtOH)/sodium acetate (NaOAc) (0.05 M) (38:62 v/v) as mobile phase, at a flow rate of 4 mL/min. The collected fraction was sterile-filtered and diluted with saline to decrease the EtOH concentration below 10%. The radiochemical purity of the produced [^11^C]CHDI-626 was greater than 99%, determined as recently described [16], with a molar activity of 189.7 ± 58.6 GBq/µmol. The tritiated version of the radioligand, namely [^3^H]CHDI-626, was synthesized by Pharmaron, UK, with radiochemical purity of 98.7% and molar activity of 3.07 GBq/µmol.

### Radiometabolite analysis

Given the rapid wash-out of the radioligand from the brain, in vivo contribution of parent fraction and radiometabolites in the plasma and brain was investigated at 5, 15, and 25 min post-injection (p.i.). Analysis was performed in both young (4 M) and aged (10 M) HET zQ175DN mice and WT littermates (*n* = 36 in total, split evenly between ages; Fig. 1) to assess age- or genotype-related differences. A bolus of [^11^C]CHDI-626 (6.4 ± 3.2 MBq, 200 μL) was injected via the lateral tail vein. Blood was withdrawn via cardiac puncture and the brain was rapidly dissected at 5, 15, and 25 min p.i. Procedure was performed adapting the previously described protocol [21, 22] to [^11^C]CHDI-626. Radiometabolite values were expressed as a percentage of the total area under the peaks detected in the reconstructed radiochromatograms.

Plasma extraction efficiencies were 86.8 ± 1.0% and 87.8 ± 2.4% for WT and HET zQ175DN mice, respectively, whereas brain extraction efficiencies were 64.7 ± 2.5% and 70.9 ± 1.7% for WT and HET zQ175DN mice, respectively. Control experiments were performed using blood and brain spiked in vitro with 37 kBq of [^11^C]CHDI-626 in order to assess radioligand stability during analytical procedure. No degradation of the radioligand occurred during analytical procedure in both plasma (WT = 99.3 ± 0.8%; HET = 99.9 ± 0.05%) and brain (WT = 98.5 ± 1.5%; HET = 99.5 ± 0.9%) samples (data not shown), indicating stability of [^11^C]CHDI-626 in plasma and brain homogenates during sample preparation and analysis.

To generate a population-based parent fraction curve, the mean measured fractions of intact radioligand at different times p.i. were fitted using a Watabe curve as this model provided the best fit compared to others (e.g. Sigmoid, Hill, or Exponentials). Thus, each image-derived input function (IDIF) was corrected for the plasma-to-whole blood ratio and the Watabe fitting for the parent fraction to derive the metabolite-corrected input function. The IDIF itself was extracted from the PET images measuring the whole blood activity in the left ventricle of the heart as previously reported [21, 23].

### Dynamic PET imaging

Dynamic microPET data were acquired in the list-mode format using two Siemens Inveon PET/CT scanners (Siemens Preclinical Solution, Knoxville, USA). Following anaesthesia with isoflurane (Forene, Belgium) supplemented in medical oxygen (induction 5%, maintenance 1.5%), animals were catheterized in the lateral tail vein for intravenous (i.v.) administration of the tracer and positioned onto the scanner bed. Physiological parameters were monitored and kept stable (respiration: 70 ± 20 breaths/min; temperature: 37 ± 1 °C) throughout the scan using a monitoring acquisition module (Minerve, France) with a feedback-controlled warm airflow. At the onset of the dynamic PET scan, a bolus injection of radioligand was performed over a 12-s interval (1 mL/min) using an automated pump (Pump 11 Elite, Harvard Apparatus, USA). Total injected mass was kept similar across time points in a trace dose (< 1.5 μg/kg), with an average injected mass of 0.60 ± 0.24 µg/kg for WT mice and 0.63 ± 0.22 µg/kg for HET zQ175DN mice. Scan parameters of the longitudinal study are reported in Table S1. Following the PET scan, a 10-min 80 kV/500 μA computed tomography (CT) scan was performed for attenuation correction and co-registration of the PET images.

Acquired PET data were reconstructed into 27 or 39 frames of increasing length (12 × 10 s, 3 × 20 s, 3 × 30 s, 3 × 60 s, 3 × 150 s, and 3 or 15 × 300 s) depending on the 30- or 90-min acquisition, respectively, using a list-mode iterative reconstruction with proprietary spatially variant resolution modelling with 8 iterations and 16 subsets of the 3D ordered subset expectation maximization (OSEM 3D) algorithm [24]. Frames were reconstructed on a 128 × 128 × 159 grid with 0.776 × 0.776 × 0.796 mm^3^ voxels with normalization, dead time, random, decay, and CT-based attenuation corrections applied.

### Image processing and analysis

Image processing and analysis were performed with PMOD 3.6 software (PMOD Technologies, Zurich, Switzerland). Spatial normalization of the PET/CT images was done through brain normalization of the CT image to the CT/magnetic resonance imaging (MRI) template adapting the previously described procedure [25]. By adjusting the volumes-of-interest (VOIs) using the Waxholm atlas [26], time-activity curves (TACs) were extracted for the striatum, motor cortex, hippocampus, and thalamus.

Regional TACs were analysed with kinetic modelling to determine the total volume of distribution (*V*_T_) using an IDIF to obtain *V*_T (IDIF)_ as its noninvasive surrogate [21, 23]. Regional TACs were fitted both by standard two-tissue compartmental model (2TCM), with blood volume fraction (*V*_B_) fixed at 3.6%, and by the Logan plot [27]. For the Logan plot, the linear phase (t*) was assessed from the curve fitting to be 1.66–2.66 min depending on the brain region. Goodness-to-fit of 2TCM based on 90-min acquisition using IDIF with or without correction for plasma metabolites was compared using the Akaike information criterion (AIC) [28], where the model with the lowest value is the preferred. Accordingly, the 2TCM with uncorrected IDIF was slightly preferable over the corrected IDIF (corrected, AIC = 82.2 ± 17.7; uncorrected, AIC = 80.8 ± 24.9). Thus, the whole blood IDIF was applied for kinetic modelling as the population-based metabolite correction did not improve the 2TCM fitting.

To determine the stability of the *V*_T (IDIF)_ estimation, *V*_T (IDIF)_ values were calculated by repeatedly shortening PET acquisition from 90 min down to 15 min, with the 90-min acquisition as a reference outcome. *V*_T (IDIF)_ estimation was considered acceptable when the average percentage difference was lower than 10%. Following kinetic analysis, Logan plot based on a 15-min acquisition with uncorrected IDIF was selected for the longitudinal study. Although 15 min is an uncommonly short acquisition for dynamic PET imaging, it was selected in large part due to the fast metabolism and wash-out of the tracer (see “Discussion”). Parametric *V*_T (IDIF)_ maps were generated through voxel-wise graphical analysis (Logan) using the uncorrected IDIF as input function and represented as averages onto the Waxholm template for anatomical reference. Signal in olfactory bulb was affected by spill-in radioactivity from the Harderian glands (Fig. S1) in both WT and HET mice; therefore, this region could not be quantified.

### Tissue collection

Cross-sectional cohorts of animals at 3, 6, and 9 M, as well as the final time point at 13 M with the animals of the longitudinal study (Fig. 1), were euthanized by decapitation while under anaesthesia. Brains were dissected and fresh-frozen in 2-metylbuthane at − 35 °C for 2 min and further preserved at − 80 °C until use. For post-mortem analysis, 20-µm-thick coronal sections were collected at 1.10 mm and − 1.46 mm from the bregma (striatal and hippocampal sections, respectively) according to Paxinos and Franklin [29] in triplicate using a cryostat (Leica, Germany) on Superfrost Plus slides (Thermo Fischer Scientific, USA).

### Autoradiography

[^3^H]CHDI-626 was performed to confirm the in vivo binding. Briefly, adjacent sections were air-dried and preincubated for 20 min in binding buffer (50 mM Tris–HCl + 120 mM NaCl + 5 mM KCl + 2 mM CaCl_2_ + 1 mM MgCl_2_, pH 7.4). Then, slides were dried using a warm airflow and incubated with the total binding solution (0.5 nM of [^3^H]CHDI-626 in binding buffer) or non-specific binding solution (0.5 nM of [^3^H]CHDI-626 + 1 µM of cold CHDI-626 in binding buffer) for 60 min at room temperature. After three 10-min washes in 50 mM Tris–HCl buffer at 4 °C and rinsed in ice-cold water, sections were dried at room temperature for 2 h. Finally, they were exposed on imaging plates (BAS-TR2025, Fujifilm, Japan) for 120 h together with a commercial tritium activity standard (American Radiolabeled Chemicals Inc., USA) for quantification of the radioactivity based on intensity values imaged using a phosphor imager (Fuji FLA-700 image reader). The measured grey intensity was transformed into radioactivity based on intensity values obtained using tritium standards with a known Bq/mg scale. Thus, based on the known decay-corrected molar activity of [^3^H]CHDI-626 (GBq/µmol) and tritium standards (Bq/mg) on the experimental day, the calculated radioactivity was transformed into fmol/mg. Specific binding in the striatum, motor cortex, hippocampus, and thalamus was calculated as the difference between the total binding and non-specific binding. All regions were delineated manually and bilaterally using Fiji ImageJ (National Institute of Health, USA). The average of each animal (in triplicate) was used for statistical analysis.

### Statistical analysis

According to the Shapiro–Wilk test, in vivo data were normally distributed, while autoradiography data were not. Comparison of striatal *V*_T (IDIF)_ values from the evaluation cohort was performed using an unpaired *t* test, while Pearson’s correlation tests were applied to correlate *V*_T (IDIF)_ values obtained with different scan acquisitions. Longitudinal in vivo PET data were analysed using a mixed-effect model with the restricted maximum likelihood method (genotype and region as variables). Post hoc multiple comparisons correction was performed with the Tukey test. Two-way ANOVA with the post hoc Bonferroni correction for multiple comparisons was applied to investigate differences between genotypes for autoradiography. Statistical analyses were performed with GraphPad Prism (version 9.1.2) statistical software. Sample size calculations at desired anticipated therapeutic effects were performed with G*Power software (version 3.1) (http://www.gpower.hhu.de/). Data are represented as mean ± standard deviation (SD). All tests were two-tailed and statistical significance was set at *p* < 0.05.

## Results

### [^11^C]CHDI-626 displays rapid metabolism and kinetics

**Fig. 2:**
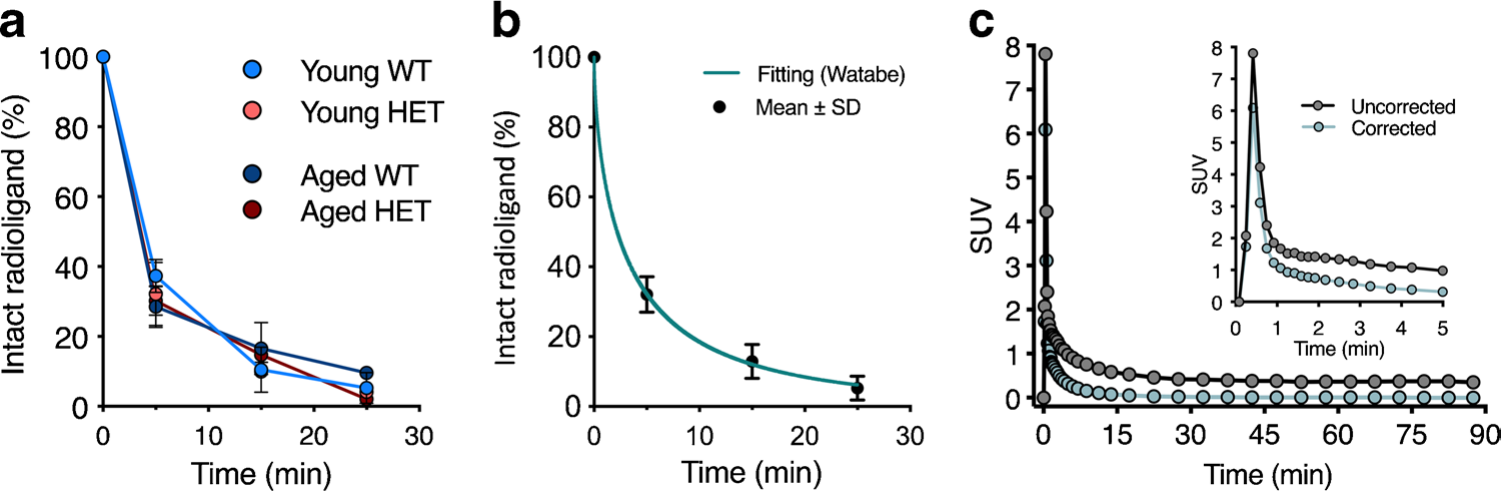
Plasma profile of [^11^C]CHDI-626. **a** Plasma profile of intact radioligand in young (early symptomatic with small mHTT aggregates detectable) and aged (symptomatic with large mHTT aggregates detectable) HET zQ175DN mice as well as WT littermates. **b** Population-based curve fitting of intact radioligand. **c** Representative 90-min image-derived input function corrected and uncorrected for the population-based metabolite profile

Plasma clearance of [^11^C]CHDI-626 was very rapid, with nearly complete disappearance at 25 min p.i. (parent fraction of 5.2 ± 1.8%) independently of age (4 M and 10 M) or genotype (WT and HET) (Fig. 2a). Radiochromatograms of plasma samples for each individual group are shown in Fig. S2. Mean values of intact radioligand at the different times p.i. were fitted by a Watabe curve (Fig. 2b) to obtain the metabolite-corrected IDIF (Fig. 2c). As a result of the rapid clearance, the metabolite-corrected IDIF approached zero already at 15 min p.i. (SUV = 0.05).

Radiochromatograms of brain samples for each individual group are shown in Fig. S3. [^11^C]CHDI-626 contribution to the brain signal appeared to be dependent on the mHTT load following the initial fast wash-out. Accordingly, at 5 min p.i. there was no difference among groups; however, [^11^C]CHDI-626 contribution was higher in HET mice at 25 min p.i. (Fig. S4).

**Fig. 3:**
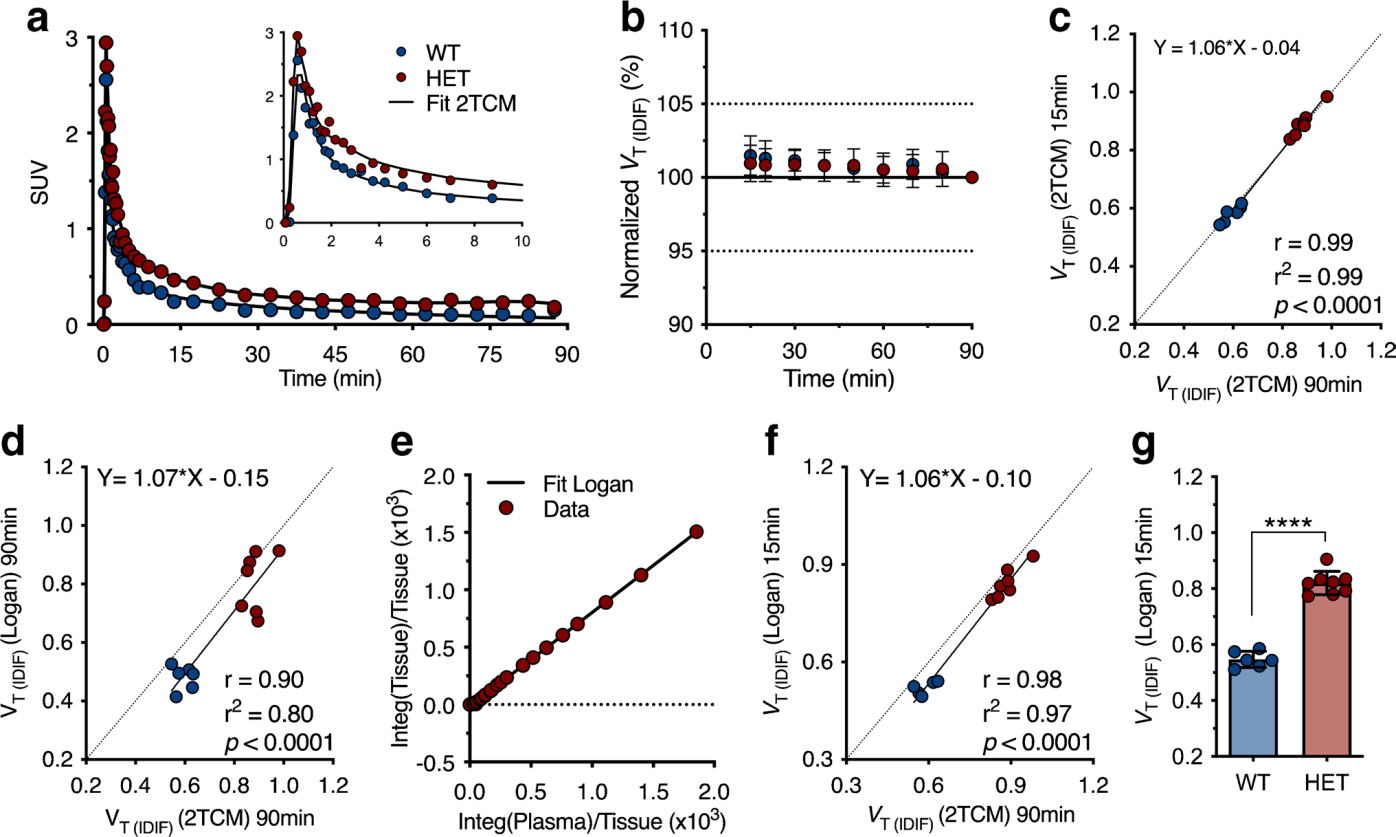
Kinetic modelling of [^11^C]CHDI-626. **a** Representative striatal SUV curves of one wild-type and one heterozygous with the curve fitting of the two-tissue compartment model (2TCM). **b** Time stability of striatal *V*_T (IDIF)_ calculated using 2TCM normalized to the values obtained with a 90-min acquisition. **c** Correlation of striatal *V*_T (IDIF)_ values calculated using 2TCM based on 90-min and 15-min acquisitions. **d** Correlation of striatal values obtained with Logan plot (90 min) compared to 2TCM (90 min) indicated the former was not sufficiently accurate to recapitulate 2TCM estimates. **e** Representative Logan plot fitting based on 15 min. **f** Correlation of striatal values calculated using Logan plot (15 min) compared to 2TCM (90 min) revealed Logan plot (15 min) to be appropriate for *V*_T (IDIF)_ estimation given the robust comparison with 2TCM values. **g** *V*_T (IDIF)_ values based on Logan and 15-min PET acquisition in WT (*n* = 6) and HET (*n* = 8) zQ175DN mice

Next, radioligand kinetics were assessed based on a dynamic 90-min [^11^C]CHDI-626 PET scan in 10 M WT and HET zQ175DN mice. Representative 2TCM fitting curves over striatal SUV TACs for WT and HET animals are shown in Fig. 3a. Radioligand penetrated rapidly into the brain, reaching the peak within 1 min p.i. in both genotypes, followed by a fast wash-out, which was less prominent in HET mice indicating binding to mHTT. Within 30 min after injection, the SUV TACs for both genotypes were plateauing, with HET mice maintaining higher values (Fig. 3a).

Given the prominent peripheral clearance and the fast brain wash-out, *V*_T (IDIF)_ could reliably be estimated with a scan duration as short as 15 min (Fig. 3b), with only a negligible deviation compared to the reference 90 min (0.9 ± 1.2% and 1.5 ± 1.3% for HET and WT mice, respectively). Accordingly, comparison of the *V*_T (IDIF)_ values based on 90- and 15-min acquisitions resulted in an excellent agreement (*r*^2^ = 0.99, *p* < 0.0001) with negligible deviation from the identity line (slope = 1.06, intercept =  − 0.04) (Fig. 3c). When investigating the suitability of the Logan plot to estimate the *V*_T (IDIF)_, the model fitted poorly when applied to 90 min of data acquisition (*r*^2^ = 0.80) (Fig. 3d) due to the fast kinetics; however, it provided a good fit for 15-min data acquisition (Fig. 3e) and the resulting values were in line with the *V*_T (IDIF)_ 2TCM values based on 90 min (*r*^2^ = 0.97, *p* < 0.0001) (Fig. 3f). Thus, Logan plot based on a 15-min acquisition was a valid alternative. Striatal *V*_T (IDIF)_ values calculated with Logan plot considering 15-min acquisition in 10 M animals displayed higher values for HET mice compared to WT littermates (+ 50 ± 3.4%, *p* < 0.0001) (Fig. 3f), highlighting the capability of [^11^C]CHDI-626 in discriminating between genotypes given the differences in target expression (i.e. mHTT aggregate load expressed in HET but not WT mice; also see the next section).

### [^11^C]CHDI-626 PET imaging detects mHTT accumulation

**Fig. 4:**
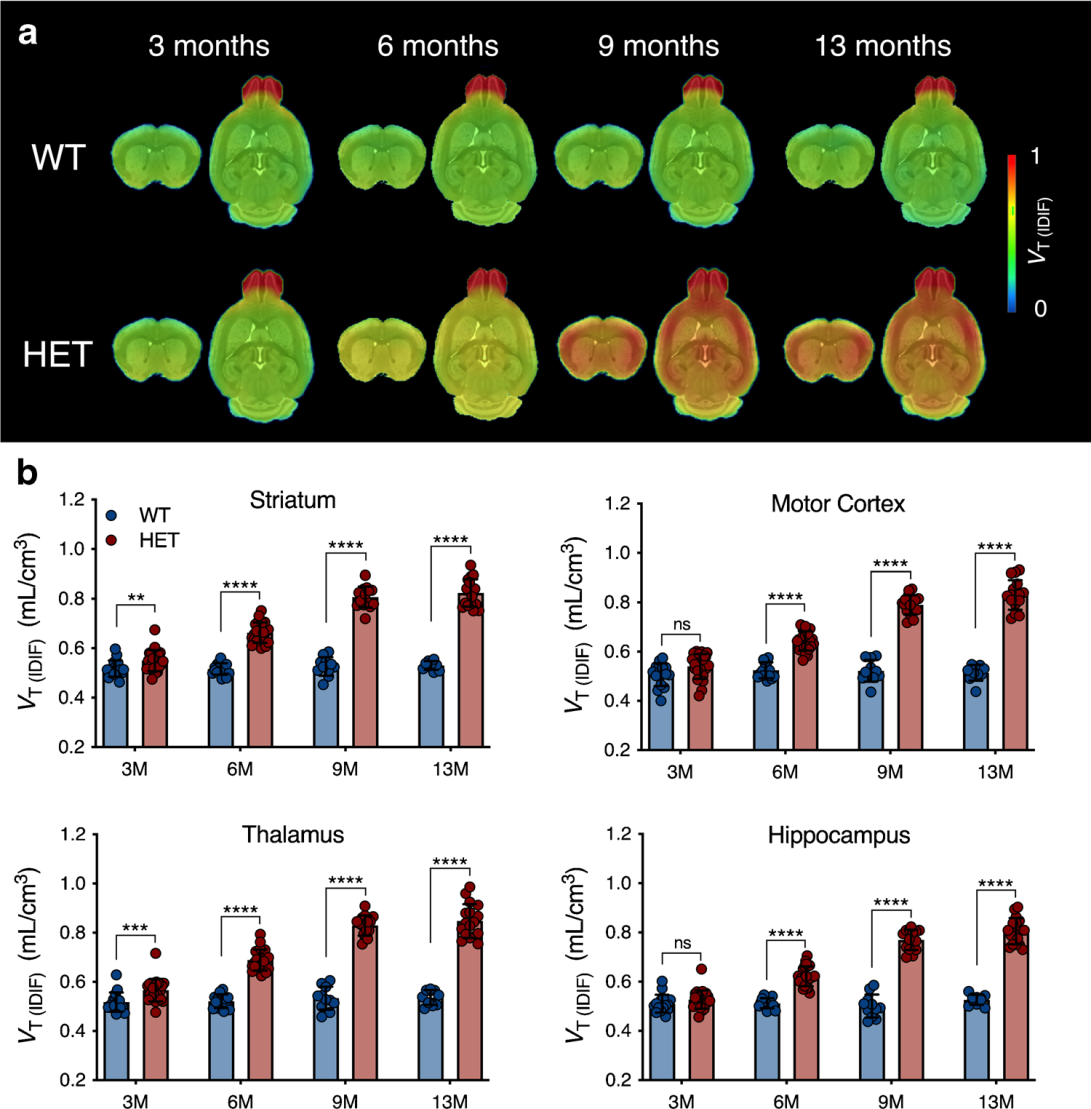
Longitudinal [^11^C]CHDI-626 PET imaging. **a** Average *V*_T (IDIF)_ parametric maps of [^11^C]CHDI-626 in HET zQ175DN mice and WT littermates. **b** Regional *V*_T (IDIF)_ quantification resulted in an age-dependent increase in [^11^C]CHDI-626 uptake in HET zQ175DN mice, while no difference in WT mice was found

Mean [^11^C]CHDI-626 *V*_T (IDIF)_ parametric maps of WT and HET mice at each investigated time point are reported in Fig. 4a. Genotypic difference was measured in all investigated brain structures (striatum: *F*_(1,122)_ = 670.7; *p* < 0.0001; motor cortex: *F*_(1,122)_ = 425.1; *p* < 0.0001; thalamus: *F*_(1,122)_ = 618.7; *p* < 0.0001; and hippocampus: *F*_(1,122)_ = 560.4; *p* < 0.0001). Notably, a significant increased [^11^C]CHDI-626 was detectable in the striatum already at 3 M (+ 6.3 ± 2.1%, *p* < 0.01) and thalamus (+ 9.3 ± 2.3%, *p* < 0.001) of HET mice. As of 6 M, all brain regions displayed a significant increase in HET mice compared to WT littermates (e.g. striatum: + 28.4 ± 2.2%, *p* < 0.0001; + 53.4 ± 3.9%, *p* < 0.0001; + 55.2 ± 3.4%, *p* < 0.0001 at 6, 9, and 13 M, respectively) (Fig. 4b; Table S2).

Temporal quantification of [^11^C]CHDI-626 PET imaging identified significant differences (striatum: *F*_(3,122)_ = 89.7, *p* < 0.0001; motor cortex: *F*_(3,122)_ = 79.0, *p* < 0.0001; thalamus: *F*_(3,122)_ = 75.3, *p* < 0.0001; and hippocampus: *F*_(3,122)_ = 88.6, *p* < 0.0001). This was related to a progressive increase in all brain regions of HET mice paralleling disease progression. For instance, striatal binding in HET mice increased to + 20.5 ± 2.1%, + 46.7 ± 2.4%, and + 49.6 ± 2.3% at 6, 9, and 13 M respectively compared to 3 M (*p* < 0.0001) (Fig. 4b; Table S2). No apparent change was visible in WT littermates, with striatal [^11^C]CHDI-626 *V*_T (IDIF)_ values varying ± 2.5% at 6, 9, and 13 M compared to 3 M.

In order to assess the potential of [^11^C]CHDI-626 PET imaging as a tool to evaluate the efficacy of mHTT-lowering therapies, we performed sample size calculations at desired anticipated effects for the design of preclinical trials with striatal [^11^C]CHDI-626 PET imaging as the primary endpoint. As shown in Table 1, given the marked genotypic difference between HET and WT littermates, a sample size of only 5 subjects would be sufficient to detect a 50% therapeutic effect at 90% confidence level in 6 M zQ175DN mice.

**Table 1:** Sample size calculations at desired therapeutic effects for the design of disease-modifying interventions using striatal [^11^C]CHDI-626 PET imaging as the endpoint

Therapeutic effect (%)	Sample size required per experimental arm (*n*)							
	3 months		6 months		9 months		13 months	
	(1-β) = 0.80	(1-*β*) = 0.90	(1-*β*) = 0.80	(1-*β*) = 0.90	(1-*β*) = 0.80	(1-*β*) = 0.90	(1-*β*) = 0.80	(1-*β*) = 0.90
100%	*n* = 16	*n* = 22	*n* = 3	*n* = 3	*n* = 3	*n* = 3	*n* = 3	*n* = 3
90%	*n* = 20	*n* = 27	*n* = 3	*n* = 3	*n* = 3	*n* = 3	*n* = 3	*n* = 3
80%	*n* = 25	*n* = 34	*n* = 3	*n* = 3	*n* = 3	*n* = 3	*n* = 3	*n* = 3
70%	*n* = 32	*n* = 44	*n* = 3	*n* = 3	*n* = 3	*n* = 3	*n* = 3	*n* = 3
60%	*n* = 44	*n* = 60	*n* = 3	*n* = 4	*n* = 3	*n* = 3	*n* = 3	*n* = 3
50%	*n* = 62	*n* = 86	*n* = 4	*n* = 5	*n* = 3	*n* = 3	*n* = 3	*n* = 3
40%	*n* = 97	*n* = 133	*n* = 6	*n* = 8	*n* = 3	*n* = 4	*n* = 3	*n* = 4
30%	*n* = 175	*n* = 242	*n* = 9	*n* = 12	*n* = 4	*n* = 5	*n* = 5	*n* = 6
20%	*n* = 383	*n* = 530	*n* = 20	*n* = 27	*n* = 8	*n* = 10	*n* = 9	*n* = 12

Values are determined based on a one-tailed test, with α = 0.05 and different (1-β) powers

### [^3^H]CHDI-626 autoradiography confirms in vivo [^11^C]CHDI-626 PET imaging

**Fig. 5:**
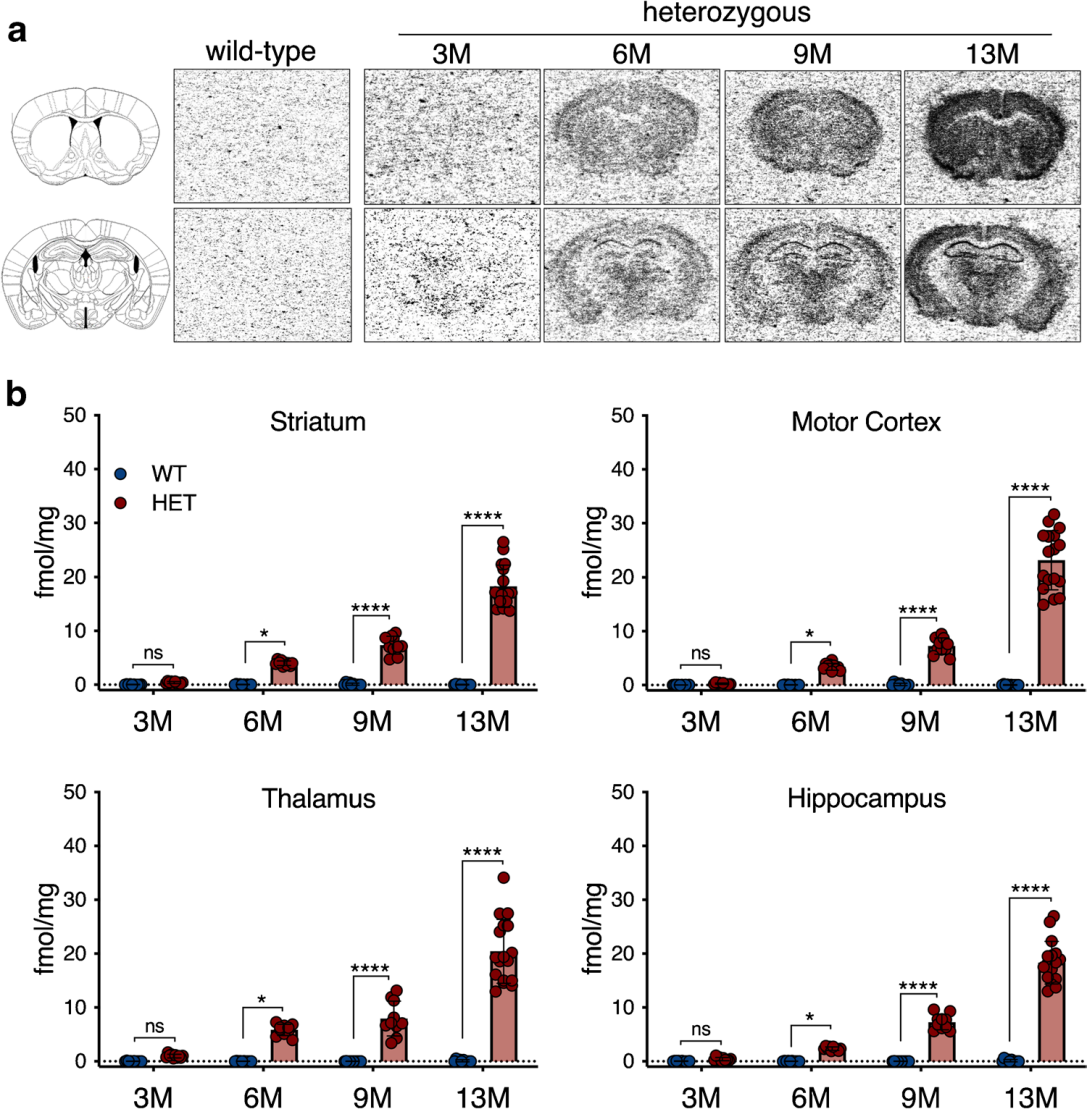
In vitro [^3^H]CHDI-626 autoradiography. **a** Representative striatal and hippocampal autoradiograms of [^3^H]CHDI-626 total binding. **b** [^3^H]CHDI-626 specific binding displayed a significant temporal increase in HET mice

Representative [^3^H]CHDI-626 autoradiograms are reported in Fig. 5a. Specific regional binding in HET mice significantly increased in parallel with the progression of the disease, while no specific signal was visible in age-matched WT littermates (striatum: *F*_(1,79)_ = 343.8, *p* < 0.0001; motor cortex: *F*_(1,79)_ = 236.5, *p* < 0.0001; thalamus: *F*_(1,79)_ = 192.5, *p* < 0.0001; and hippocampus: *F*_(1,79)_ = 306.7, *p* < 0.0001) (Fig. 5b). Noteworthy, striatal genotypic differences were significant only starting at 6 M (e.g. striatum: HET = 4.00 ± 0.4 fmol/mg, WT = 0.03 ± 0.1 fmol/mg, *p* < 0.001), although at 3 M, higher specific binding could be detected in HET mice compared to WT, albeit not statistically significant (HET = 0.41 ± 0.2 fmol/mg, wild-type = 0.02 ± 0.1 fmol/mg, *p* > 0.99) (Fig. 5b).

## Discussion

We report here the kinetic properties and longitudinal quantification of the novel radioligand [^11^C]CHDI-626 for PET imaging of mHTT in the living brain.

Based on our recent report [16], we first assessed the stability and kinetic properties of the radioligand to determine the optimal protocol for the longitudinal study. Plasma analysis revealed a rapid disappearance of [^11^C]CHDI-626 with the detection of at least 2 species of metabolites that were polar and appeared not to penetrate the blood–brain barrier as previously suggested [16]. The radioligand almost completely disappeared by 25 min, apparently due to metabolism, and its contribution to the input function was nearly absent at 15 min following injection. On the other hand, brain analysis suggested the total contribution of intact radioligand was dependent on genotype and age. As there is no evidence for significant brain-specific metabolism [16], the presence of cerebral metabolites is likely to be due to both the rapid plasma clearance and the fast wash-out of the radioligand from the brain (SUV was only 0.2 in WT mice at 15 min p.i.). With the blood fraction of the brain estimated around 3.6–4% [30] primarily composed of metabolites, they will be a key contributor to the total activity extracted from the brain. Accordingly, no difference was detected among ages or genotypes at 5 min p.i.. However, at later times (10–15 min p.i.), radioligand wash-out was dominated by mHTT load in the brain; therefore, the difference in intact radioligand in the brain between genotypes and within HET mice was a function of mHTT aggregate load.

Kinetic analysis indicated 2TCM as the most suitable model to estimate [^11^C]CHDI-626 *V*_T (IDIF)_. Because of the radioligand rapid clearance and short retention in the brain, *V*_T (IDIF)_ could be estimated with a scan as short as 15 min. Although kinetic modelling on 15-min dynamic acquisition is rather unusual, a marked difference with low variability in [^11^C]CHDI-626 binding was observed between HET and WT mice.

After optimizing PET imaging parameters, we performed a longitudinal study to monitor the temporal changes of mHTT levels in HET mice at various stages of the disease progression and compared them to WT littermates. Longitudinal [^11^C]CHDI-626 PET imaging showed a significant progressive increase in radioligand binding in HET mice.

Accumulation of mHTT, and consequently radioligand binding, was visible with both in vivo and in vitro techniques, and in agreement with previous histological analysis [20].

Importantly, *V*_T (IDIF)_ values in wild-type mice did not show any change over time or across brain regions, indicating the expected lack of specific binding as well as lack of brain-penetrant metabolites. As a result of the measured significant genotypic difference, sample size calculations showed that only 12 animals per treatment are required at 6 M to detect an adequately powered 30% therapeutic effect. While this is already striking, only 6 mice per treatment arm would be required at 9 M. Collectively, these observations suggest [^11^C]CHDI-626 PET imaging can serve as a marker to monitor disease progression.

[^11^C]CHDI-626 PET imaging revealed a significantly increased binding in HET compared to WT mice as early as 3 M, an early pre-symptomatic time point during which only very small mHTT aggregates are visible histologically [20]. In contrast, in vitro [^3^H]CHDI-626 autoradiography displayed higher values in HET compared to WT at 3 M but failed to detect differences. This observation could indicate that in vivo PET imaging provides higher sensitivity than in vitro autoradiography in detecting differences between genotypes at 3 M, a relevant aspect for translation to clinical settings. Indeed, the in vitro profile of CHDI-626 suggests its potential as an mHTT PET radioligand in humans [16], particularly due to its lack of affinity towards amyloid plaques and/or tau tangles, as previously observed in human tissue with other mHTT-directed radioligands [15, 17]. However, the rapid clearance and kinetics observed represent a limitation for [^11^C]CHDI-626 PET imaging in detecting more subtle changes in mHTT load in mice, exemplified by the in vivo vs in vitro dichotomy between 9 and 13 M in HET mice. Accordingly, [^3^H]CHDI-626 autoradiography could demonstrate an increased binding in HET mice, in agreement with histological findings [20]. Nonetheless, in larger animal models and humans, the application of individual metabolite-corrected plasma input function and serial blood sampling might offer increased sensitivity for [^11^C]CHDI-626. Clinical evaluation of this radioligand is currently underway.

## Supplementary Information

Below is the link to the electronic supplementary material.

Supplementary file1 (DOCX 9717 KB)

## Data Availability

All requests for data will be promptly reviewed by the institutions involved to verify whether the request is subject to any intellectual property or confidentiality obligations. If deemed necessary, a material transfer agreement between the requestor and institutions involved may be required for sharing of some data. Any data that can be freely shared will be released.
