# Supplementary material for: Longitudinal preclinical evaluation of the novel radioligand [11C]CHDI-626 for PET imaging of mutant huntingtin aggregates in Huntington’s disease
Source: Eur J Nucl Med Mol Imaging. 2021 Oct 15;49(4):1166–75. doi: 10.1007/s00259-021-05578-8 (PMC8921134; doi:10.1007/s00259-021-05578-8)
Supplement: Supplementary file 1 — Supplementary file1 (DOCX 9717 KB) [file 259_2021_5578_MOESM1_ESM.docx]

**SUPPLEMENTARY INFORMATION**

**Title: Longitudinal preclinical evaluation of the novel radioligand [^11^C]CHDI-626 for PET imaging of mutant huntingtin aggregates in Huntington’s Disease**

**Journal:** European Journal of Nuclear Medicine and Molecular Imaging

**Authors:** Daniele Bertoglio^1,2^, Jeroen Verhaeghe^1^, Alan Miranda^1^, Leonie wyffels^1,3^, Sigrid Stroobants^1,2,3^, Ladislav Mrzljak^4^, Vinod Khetarpal^4^, Mette Skinbjerg^4^, Longbin Liu^4^, Celia Dominguez^4^, Ignacio Munoz-Sanjuan^4^, Jonathan Bard^4^, Steven Staelens^1,2,*^

^1^Molecular Imaging Center Antwerp (MICA), University of Antwerp, Wilrijk, Belum

^2.^ μNEURO Research Centre of Excellence, University of Antwerp, Antwerp, Belgium

^3^Department of Nuclear Medicine, Antwerp University Hospital, Edegem, Belgium

^4^CHDI Management/CHDI Foundation, Los Angeles, CA, USA

***Corresponding author**

Prof. Dr. Steven Staelens

MICA, University of Antwerp, Universiteitsplein 1, Wilrijk, Belgium

Tel. +32 03265 2820

Email: [steven.staelens@uantwerpen.be](mailto:steven.staelens@uantwerpen.be)

Daniele Bertoglio, PhD

MICA, University of Antwerp, Universiteitsplein 1, Wilrijk, Belgium

Tel. +32 032652816; Email: [daniele.bertoglio@uantwerpen.be](mailto:daniele.bertoglio@uantwerpen.be)

Orcid: <https://orcid.org/0000-0003-4205-5432>

**Short running title:** Imaging mHTT in Huntington’s Disease

**Supplementary Information**


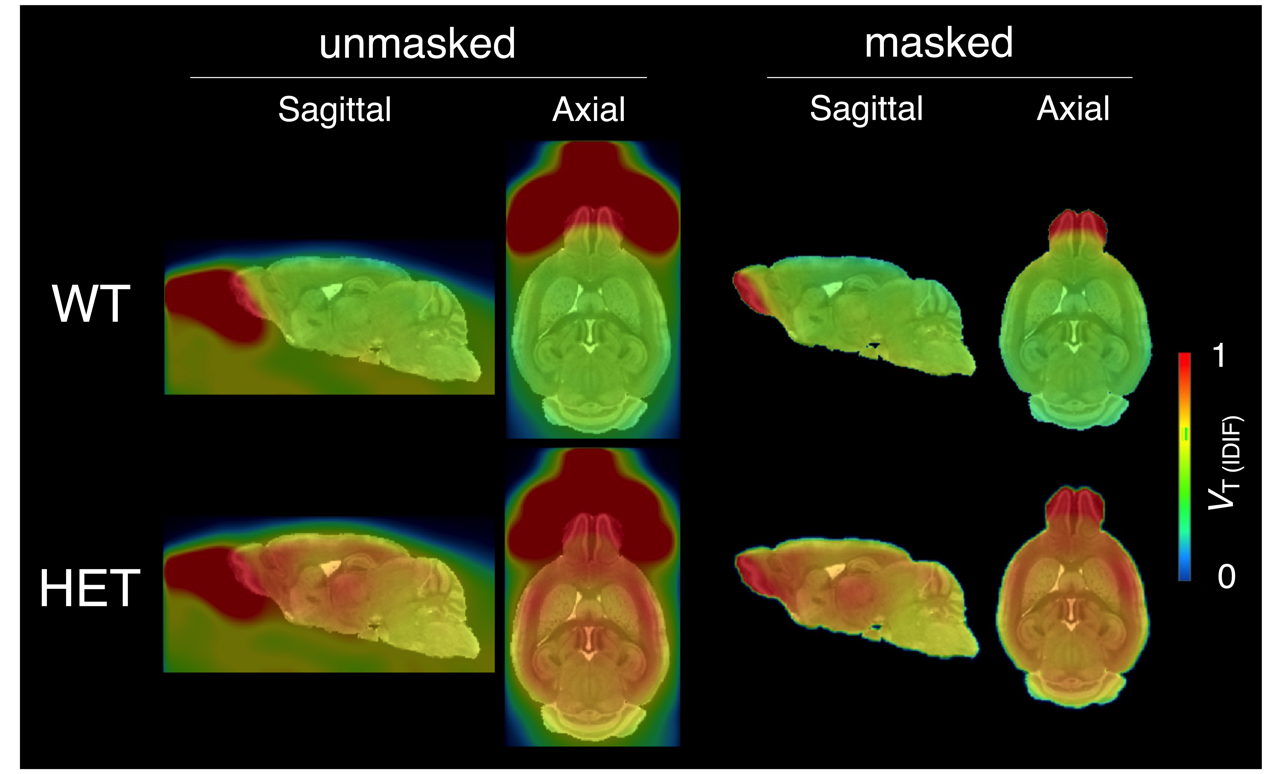


**Fig. S1** Brain masked and unmasked average *V*_T (IDIF)_ parametric maps of [^11^C]CHDI-626 in HET zQ175DN mice and WT littermates at 13M showing the spill-in into olfactory bulb caused by the radioactivity in Harderian glands.

**
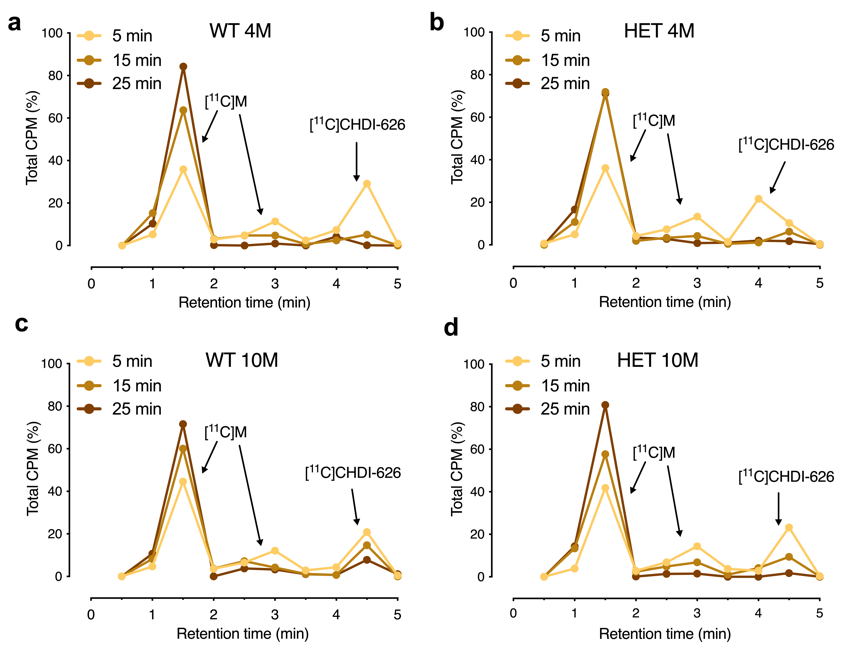
**

**Fig. S2** [^11^C]CHDI-626 plasma radiochromatograms at 5, 15, and 25 min post-injection (p.i.). Both young and aged WT **a** and HET **b** mice displayed a comparable rapid metabolism of the radioligand following intravenous injection with the formation of at least 2 species of radiometabolites ([^11^C]M). Data are presented as % of area per collected sample (mean) in the radiochromatogram

**
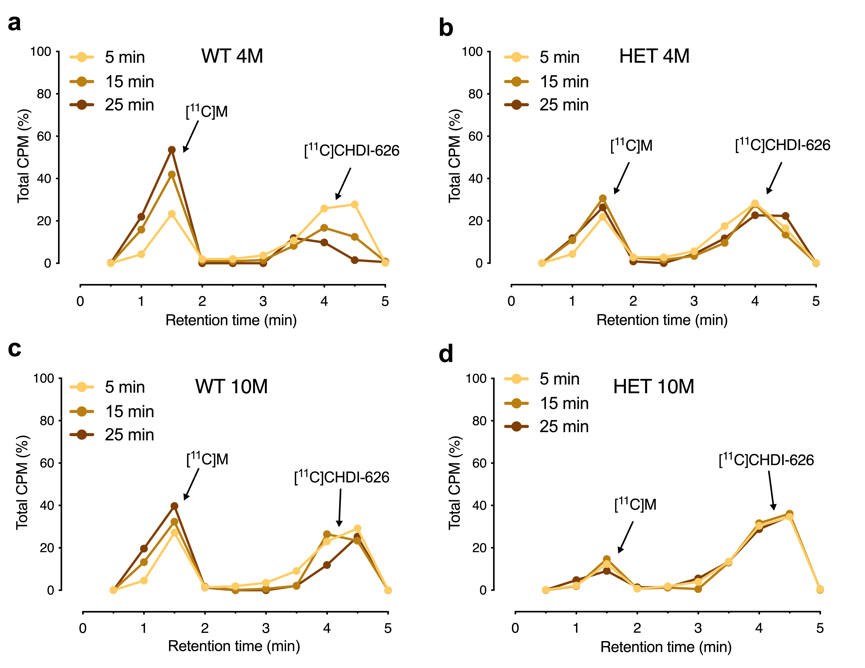
**

**Fig. S3** [^11^C]CHDI-626 brain radiochromatograms at 5, 15, and 25 min post-injection (p.i.). Following intravenous injection, both young **a** and aged **c** wild-type mice displayed a comparable profile with increasing contribution over time of radiometabolites ([^11^C]M) from the blood fraction due to the rapid wash-out of the radioligand from the brain. In contrast, young **b** and aged **d** heterozygous mice displayed a profile with the stable contribution over time of radiometabolites ([^11^C]M) from blood fraction. Data are presented as % of total counts per min (mean) in the radiochromatogram


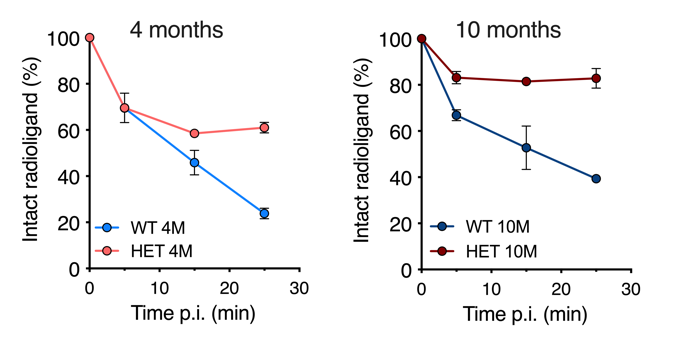


**Fig. S4** Fraction of [^11^C]CHDI-626 contributing to the total brain signal. Brain profile of intact radioligand in young (4M) and aged (10M) WT and HET zQ175DN mice appeared to be dependent on the mHTT load

**Table S1** Scan parameters during the longitudinal PET imaging study in WT and HET zQ175DN mice.

Values are expressed as mean (SD)

**Table S2** *V*_T (IDIF)_ values and genotypic difference during the longitudinal PET imaging study in WT and HET zQ175DN mice.

Values are expressed as mean (SD)
